# Supplementary material for: Pancreatic cancer intrinsic PI3Kα activity accelerates metastasis and rewires macrophage component
Source: EMBO Mol Med. 2021 May 25;13(7):e13502. doi: 10.15252/emmm.202013502 (PMC8261517; doi:10.15252/emmm.202013502)
Supplement: Supplementary file 1 — Appendix [file EMMM-13-e13502-s002.pdf]

Appendix

Table of contents

Appendix Figure S1: PI3Kα activation signature

Appendix Figure S2: Additional data on cohorts

Appendix Figure S3: All data related to western Blot of Akt

Appendix Figure S4: All data relating to migration assays

Appendix Figure S5: All data relating to cytotoxicity assays

Appendix Figure S6: GI30 and IC30 for cytotoxicity and migration experiments

Appendix Figure S7: HPNE hTERT cell cytotoxicity and migration experiments

Appendix Figure S8: PIP and PIP_2 sub-types

Appendix Figure S9: Correlations between cellular phenotypes and class I PI3K isoform inhibition

Appendix Figure S10: Additional information on cfDNA in KPC mice

Appendix Figure S11: Description of the KPC cohort

Appendix Figure S12: Tumour progression by US imaging

Appendix Figure S13: Effects of PI3Kα pharmacological inhibition on cfDNA and metastasis

Appendix Figure S14: Cancer staging and tumour histology of KPC mice treated with the vehicle and BYL-719

Appendix Figure S15: Secondary effects of PI3Kα pharmacological inhibition

Appendix Figure S16: Pharmacological inactivation of PI3Kα reverses pre-cancer lesions and alterations of their stromal environment in an oncogenic KRAS context

Appendix Figure S17: Murine cell lines derived for the study

Appendix Figure S18: Effects of PI3Kα inhibition on the immune response

Appendix additional Methods

Appendix supplemental references

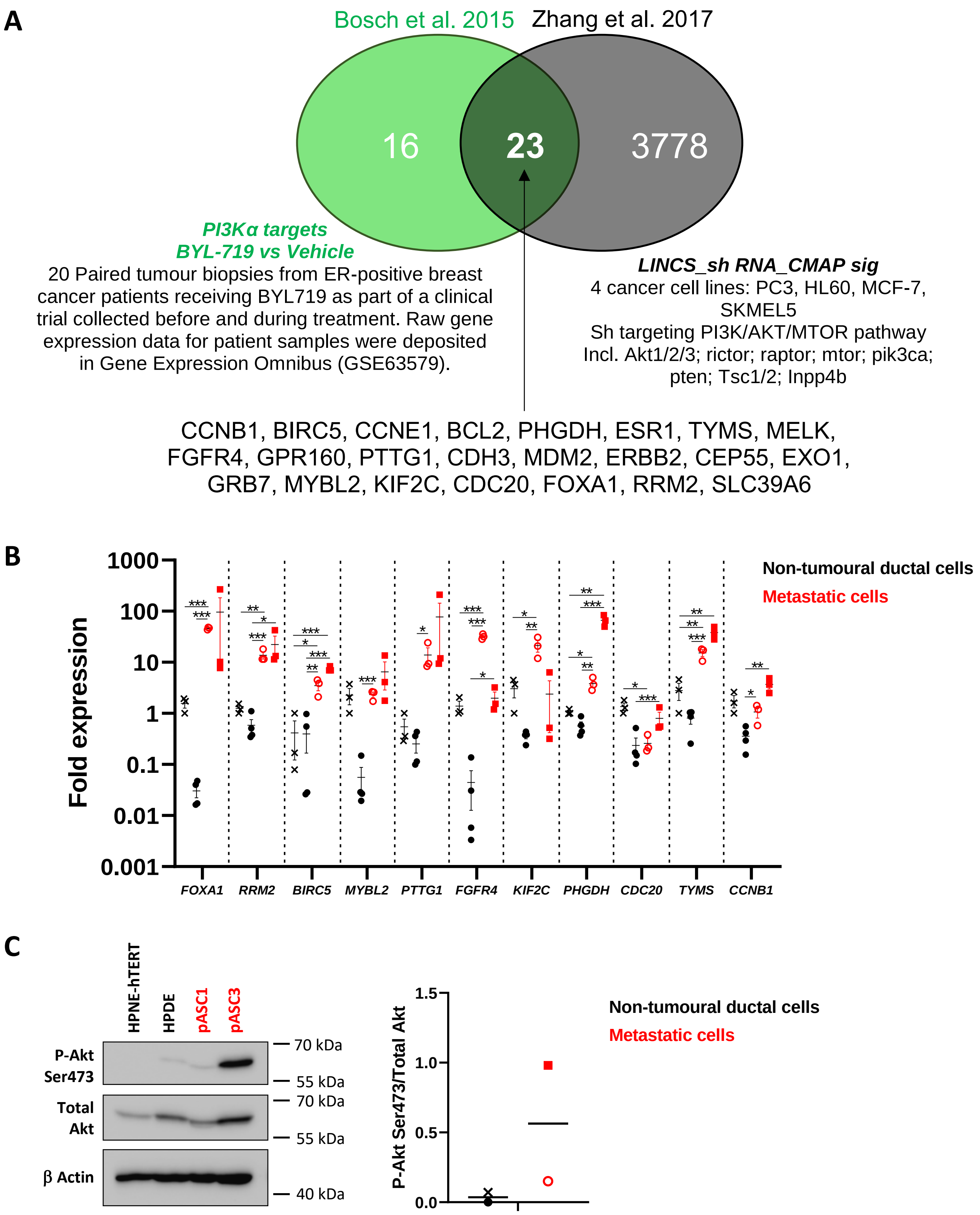
A
Bosch et al. 2015
Zhang et al. 2017
16
23
3778
PI3Kα targets
BYL-719 vs Vehicle
20 Paired tumour biopsies from ER-positive breast cancer patients receiving BYL719 as part of a clinical trial collected before and during treatment. Raw gene expression data for patient samples were deposited in Gene Expression Omnibus (GSE63579).
LINCS_sh RNA_CMAP sig
4 cancer cell lines: PC3, HL60, MCF-7, SKMEL5
Sh targeting PI3K/AKT/MTOR pathway
Incl. Akt1/2/3; rictor; raptor; mtor; pik3ca; pten; Tsc1/2; Inpp4b
CCNB1, BIRC5, CCNE1, BCL2, PHGDH, ESR1, TYMS, MELK, FGFR4, GPR160, PTTG1, CDH3, MDM2, ERBB2, CEP55, EXO1, GRB7, MYBL2, KIF2C, CDC20, FOXA1, RRM2, SLC39A6
B
Fold expression
1000
100
10
1
0.1
0.01
0.001
FOXA1
RRM2
BIRC5
MYBL2
PTTG1
FGFR4
KIF2C
PHGDH
CDC20
TYMS
CCNB1
Non-tumoural ductal cells
Metastatic cells
C
HPNE-hTERT
HPDE
pASC1
pASC3
P-Akt Ser473
Total Akt
β Actin
70 kDa
55 kDa
70 kDa
55 kDa
40 kDa
P-Akt Ser473/Total Akt
1.5
1.0
0.5
0.0
Non-tumoural ductal cells
Metastatic cells

Appendix Figure S1: PI3Kα activation signature. **A**, Identification of a PI3Kα activation signature by overlapping two distinct signatures identified by Bosch *et al.* 2015 and Zhang *et al.* 2017. **B**, Expression of *FOXA1*, *RRM2*, *BIRC5*, *MYBL2*, *PTTG1*, *FGFR4*, *KIF2C*, *PHGDH*, *CDC20*, *TYMS*, *CCNB1* (genes from PI3Kα signature) was tested by RT-qPCR in non-tumoural ductal cells (cross: HPNE hTERT, circles: HPDE) and peritoneal metastatic cells isolated from patients (empty circles: pASC1, square: pASC3). Independent replicates with the mean are represented and gene expression is relative to one replicate of AsPC-1 cells. * $p<0.05$, ** $p<0.01$, *** $p<0.001$, $n\geq3$, Student's t-test. **C**, Protein levels of P-Akt (Ser473), total Akt and β Actin were observed by western blot and P-Akt Ser473/total Akt was represented in non-tumoural ductal cells (cross: HPNE hTERT, circles: HPDE) and peritoneal metastatic cells isolated from patients (empty circles: pASC1, square: pASC3).

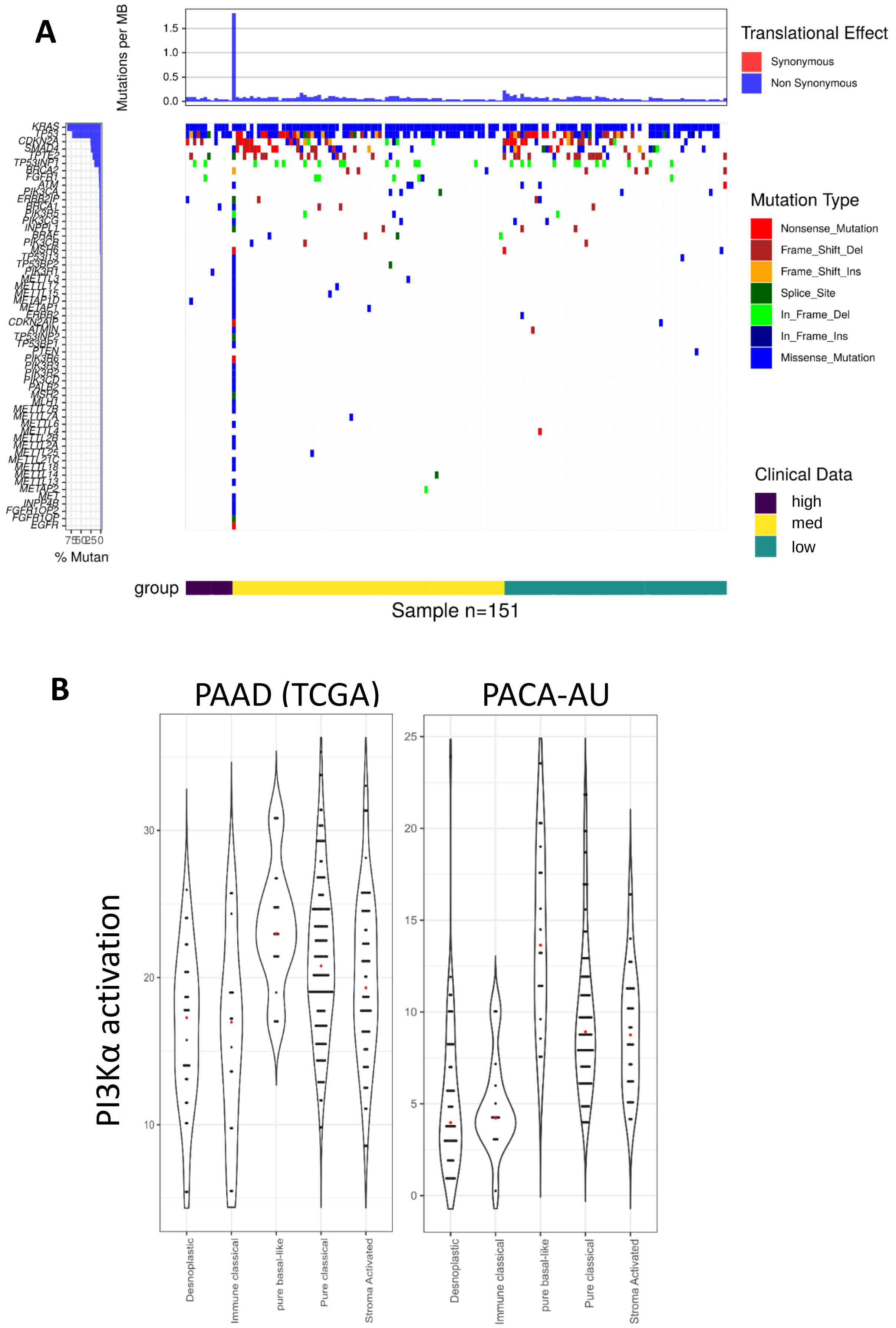

Appendix Figure S2: Additional data on cohorts. **A**, Distribution of genetic alterations in PAAD cohort (most frequent alterations and alterations related to PI3K activation). **B**, SES scores of PI3Kα activation signature in RNA based subclassification of PDAC. Origin of cohorts as indicated.

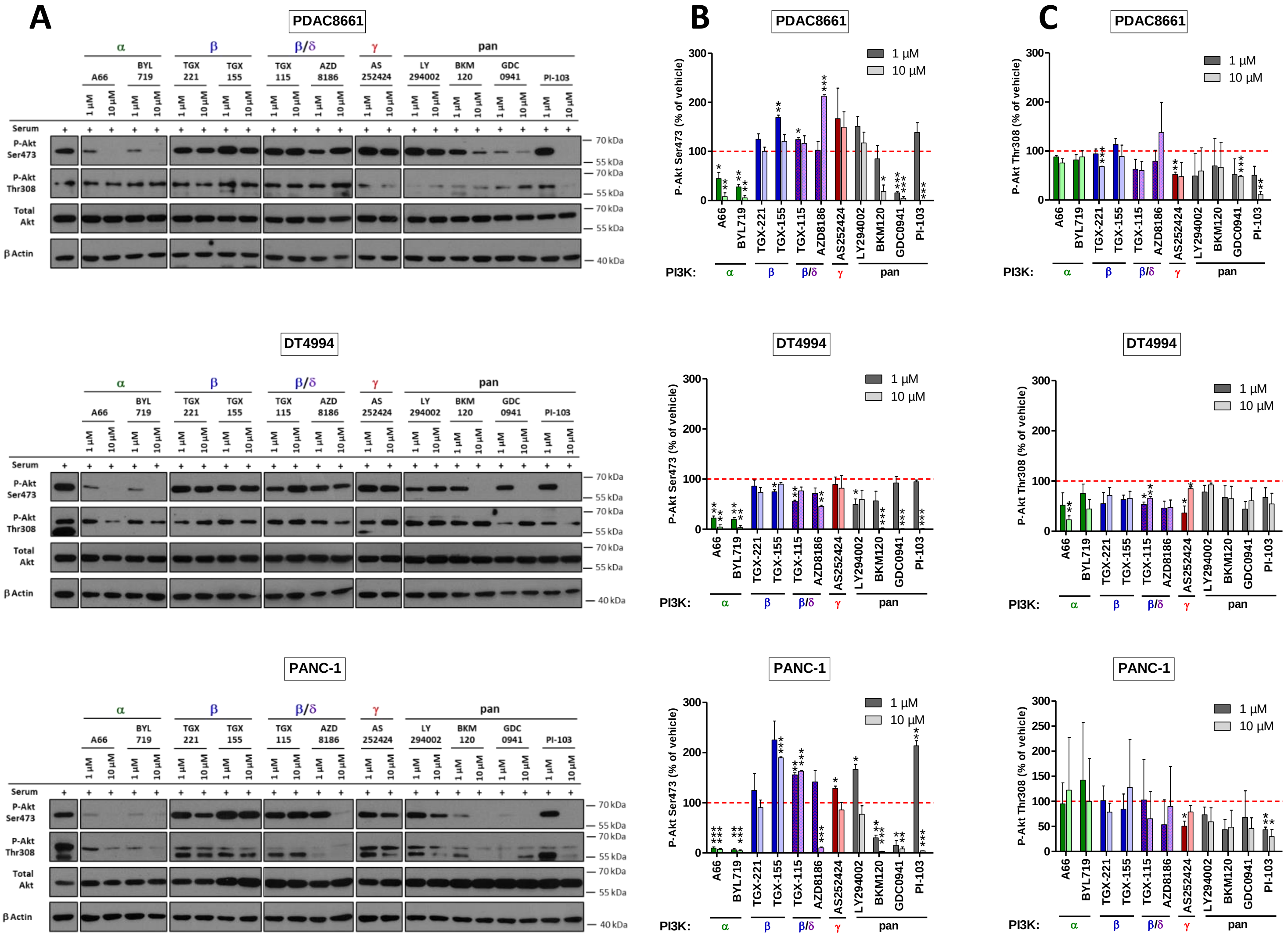

Appendix Figure S3: All data related to western Blot of Akt. A, Murine (PDAC8661, DT4994) and human (PANC-1) pancreatic tumours cells were treated for 15min with α, β, β/δ, γ-selective or pan-PI3K inhibitors at 0.1 or 1 μM in the presence of 10% FBS and the protein levels of P-Akt (Ser473 and Thr308), total Akt and β Actin were observed by western blot. P-Akt on **B**, Ser473 or **C**, Thr308 was quantified and normalised with β Actin.

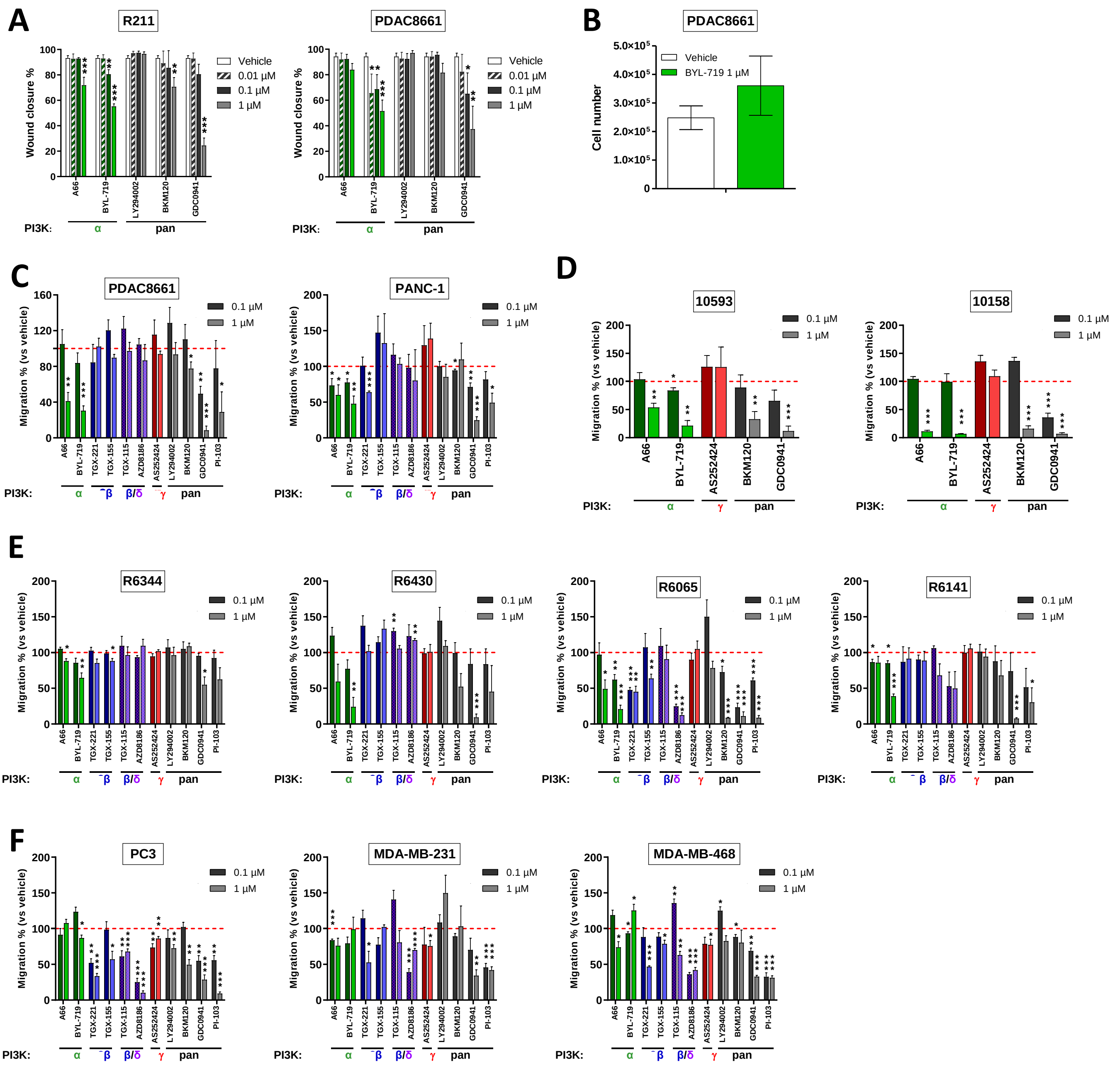

Appendix Figure S4: All data relating to migration assays. A, Scratching was applied to a monolayer of murine pancreatic tumour cells R211 or PDAC8661. The cells were treated concomitantly with α-selective or pan-PI3K inhibitors at 0.01, 0.1 or 1 µM and observed after 24 h to determine the number of wounds that had healed. **B**, murine pancreatic cells PDAC8661, whether or not treated with BYL-719 at 1 µM were counted 24 h after wound healing assay. **C**, Murine (PDAC8661) and human (PANC-1) pancreatic tumour cells were treated with α, β, β/δ, γ- selective or pan-PI3K inhibitors at 0.1 or 1 µM. **D**, KRAS^{wt}/PIK3CA^{mt} murine pancreatic tumour cells 10593 and 10158 were treated with α, γ-selective or pan-PI3K inhibitors at 0.1 or 1 µM. **E**, Murine pancreatic tumour cells (R6344, R6430, R6065, R6141) were treated with α, β, β/δ, γ- selective or pan-PI3K inhibitors at 0.1 or 1 µM. **F**, Prostate (PC3) and breast cancer cells (MDA-MB-231, MDA-MB-468) were treated with α, β, β/δ, γ- selective or pan-PI3K inhibitors at 0.1 or 1 µM. **C-F**, Cells were concomitantly subjected to a Boyden chamber migration assay and migrating cells were quantified after 24h. Mean +/- SEM (* $p<0.05$, ** $p<0.01$, *** $p<0.001$, $n\geq3$, Student's t-test).

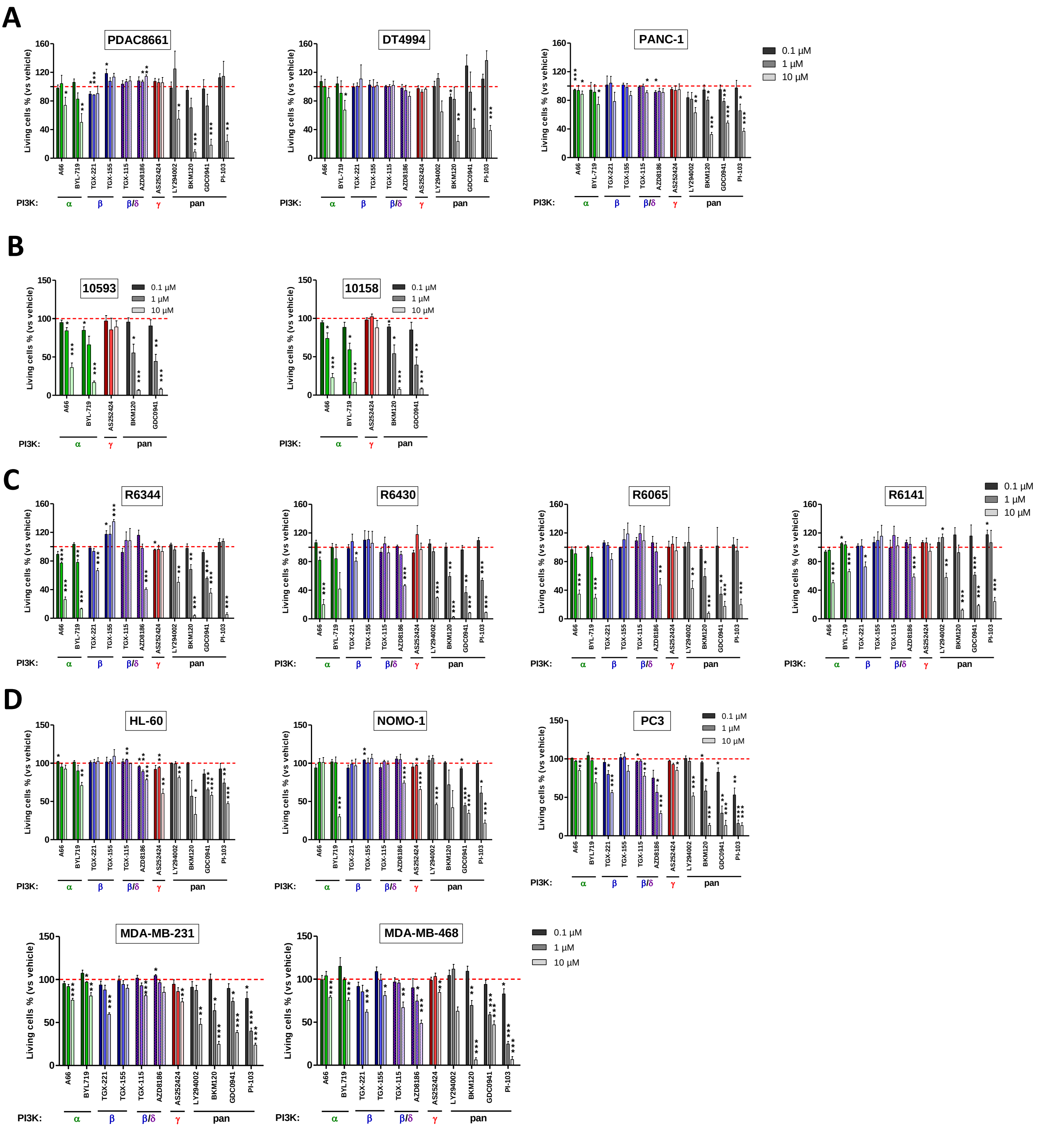

Appendix Figure S5: All data relating to cytotoxicity assays. A, Murine (PDAC8661, PDAC8661, DT4994) and human (PANC-1) pancreatic tumour cells were treated with α, β, β/δ, γ-selective or pan-PI3K inhibitors for 3 days. **B**, $KRAS^{wt}/PIK3CA^{mt}$ murine pancreatic tumour cells 10593 and 10158 were treated with α, γ-selective or pan-PI3K inhibitors. **C**, Murine pancreatic tumour cells (R6344, R6430, R6065, R6141) were treated with α, β, β/δ, γ-selective or pan-PI3K inhibitors. **D**, Acute myeloid leukaemia (HL-60, NOMO-1), prostate (PC3) and breast cancer cells (MDA-MB-231, MDA-MB-468) were treated with α, β, β/δ, γ- selective or pan-PI3K inhibitors. **A-D**, Living cells were quantified after 3 days with a MTT colorimetric assay and metabolically active cells are considered as living cells. Mean +/- SEM (* $p<0.05$, ** $p<0.01$, *** $p<0.001$, $n\geq3$, Student's t-test).

A

	Cytotoxicity - Growth GI30 (µM)											
	Cell line	A66	BYL-719	TGX-221	TGX-155	TGX-115	AZD8186	AS252424	LY294002	BKM120	GDC0941	PI-103
		α		β		β/δ		γ	pan			
Pancreas	R211	NR	5.804	NR	NR	NR	NR	NR	2.097	1.307	1.699	1.878
	PDAC8661	NR	1.867	NR	NR	NR	NR	NR	9.575	1.022	1.127	6.389
	DT4994	NR	6.082	NR	NR	NR	NR	NR	9.096	2.370	2.043	8.421
	PANC-1	NR	NR	NR	NR	NR	NR	NR	4.15	1.638	1.654	0.818
	10593	2.017	0.8107	-	-	-	-	NR	-	0.615	0.410	-
	10158	1.182	0.6293	-	-	-	-	NR	-	0.545	0.312	-
	R6344	1.443	1.311	6.663	NR	NR	2.662	NR	3.556	0.944	0.501	6.288
	R6430	1.487	1.955	NR	NR	NR	2.617	NR	2.471	0.723	0.359	0.646
	R6065	2.787	1.928	NR	NR	NR	2.887	NR	8.330	0.701	0.331	2.528
	R6141	7.519	7.608	NR	NR	NR	5.753	NR	8.477	1.845	0.782	2.965
Other organs	PC3	NR	8.671	2.005	NR	NR	0.3351	NR	4.203	0.660	0.217	0.058
	MDA-MB-231	NR	NR	3.832	NR	NR	NR	NR	3.163	0.794	1.296	0.217
	MDA-MB-468	NR	NR	3.800	NR	7.790	1.365	NR	8.549	0.989	0.513	0.206
	NOMO-1	NR	4.291	NR	NR	NR	NR	8.741	7.341	1.096	0.319	0.727
	HL-60	NR	NR	NR	NR	NR	NR	8.326	NR	0.583	0.629	1.284

	Cytotoxicity - Growth GI30 (µM)											
	Cell line	A66	BYL-719	TGX-221	TGX-155	TGX-115	AZD8186	AS252424	LY294002	BKM120	GDC0941	PI-103
		α		β		β/δ		γ	pan			
Pancreas	HPNE hTERT	3.644	4.141	NR	NR	NR	NR	NR	8.801	1.174	0.541	1.366

NR = not reached
- = non-available

B

	Migration - Inhibitory IC30 (µM)											
	Cell line	A66	BYL-719	TGX-221	TGX-155	TGX-115	AZD8186	AS252424	LY294002	BKM120	GDC0941	PI-103
		α		β		β/δ		γ	pan			
Pancreas	R211	0.045	0.044	NR	NR	NR	NR	NR	NR	NR	0.035	0.151
	PDAC8661	0.814	0.177	NR	NR	NR	NR	NR	NR	NR	0.053	0.137
	PANC-1	0.133	0.148	0.8217	NR	NR	NR	NR	NR	NR	0.105	0.187
	10593	0.854	0.171	-	-	-	-	NR	-	0.511	0.033	-
	10158	0.694	0.417	-	-	-	-	NR	-	0.918	0.038	-
	R6344	NR	0.375	NR	NR	NR	NR	NR	NR	NR	0.416	0.463
	R6430	0.986	0.132	NR	NR	NR	NR	NR	NR	0.536	0.163	0.198
	R6065	0.421	0.076	0.028	0.846	NR	0.026	NR	NR	0.109	0.026	0.075
	R6141	NR	0.198	NR	NR	0.941	0.033	NR	NR	0.604	0.114	0.048
Other organs	PC3	NR	NR	0.048	0.548	0.014	0.028	NR	NR	0.743	0.056	0.064
	MDA-MB-231	NR	NR	0.861	NR	NR	0.013	NR	NR	NR	0.101	0.030
	MDA-MB-468	NR	NR	0.252	NR	0.963	0.012	NR	NR	NR	0.095	0.021

	Migration - Inhibitory IC30 (µM)											
	Cell line	A66	BYL-719	TGX-221	TGX-155	TGX-115	AZD8186	AS252424	LY294002	BKM120	GDC0941	PI-103
		α		β		β/δ		γ	pan			
Pancreas	HPNE hTERT	0.989	NR	NR	NR	NR	0.921	NR	NR	NR	0.244	0.148

NR = not reached
- = non-available

Appendix Figure S6: GI30 and IC30 for cytotoxicity and migration experiments. A, Pancreatic cancer (R211, PDAC8661, DT4994, PANC-1, 10593, 10158, R6344, R6430, R6065, R6141), pancreatic untransformed (HPNE hTERT), prostate cancer (PC3), breast cancer cells (MDA-MB-231, MDA-MB-468) and acute myeloid leukaemia cells (NOMO-1, HL-60) were treated with 0.1, 1 or 10 µM of α, β, β/δ, γ-specific or pan-PI3K inhibitors and living cells were quantified after 3 days with a MTT colorimetric assay. GI30 (for the concentration that inhibited 30% of cell growth) was determined with GraphPad Prism 8 using log (inhibitor) vs. response - variable slope (four parameters) analysis. "-" indicates that the inhibitor was not used in the corresponding cell line, "NR" indicates that the GI30 was not reached. **B**, Pancreatic cancer (R211, PDAC8661, DT4994, PANC-1, 10593, 10158, R6344, R6430, R6065, R6141), pancreatic untransformed (HPNE hTERT), prostate cancer (PC3) and breast cancer cells (MDA-MB-231, MDA-MB-468) were treated with 0.1 or 1 µM of α, β, β/δ, γ-specific or pan-PI3K inhibitors and concomitantly subjected to a Boyden chamber migration assay. Migrating cells were quantified after 24h. CI30 (for the concentration that inhibited 30% of cell migration) was determined with GraphPad Prism 8 using log (inhibitor) vs. response - variable slope (four parameters) analysis. "-" indicates that the inhibitor was not used in the corresponding cell line, "NR" indicates that the GI30 was not reached. Mean +/- SEM (* $p<0.05$, ** $p<0.01$, *** $p<0.001$, $n\geq3$, Student's t-test).

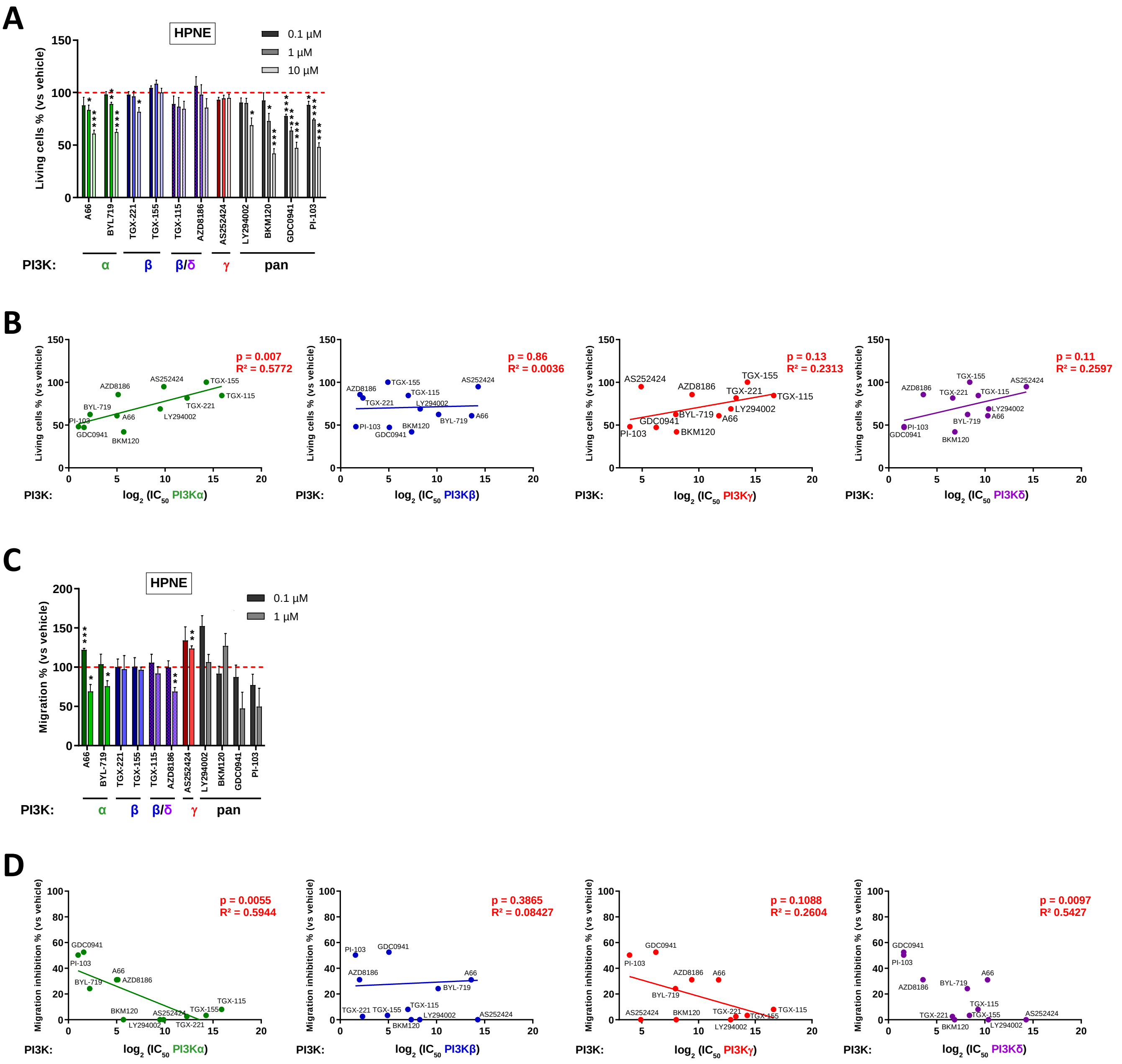

Appendix Figure S7: HPNE hTERT cell cytotoxicity and migration experiments. A, Ductal pancreatic cells HPNE hTERT were treated with α, β, β/δ, γ-specific or pan-PI3K inhibitors and living cells were quantified after 3 days with a MTT colorimetric assay. Metabolically active cells are considered as living cells. **B**, Cytotoxic capacities of PI3K inhibitors at 1 µM were tested on HPNE hTERT cells and were plotted against the in vitro IC50 for each class I PI3K of each inhibitor determined on recombinant proteins. Pearson correlation tests were performed. **C**, HPNE hTERT cells were treated with α, β, β/δ, γ-specific or pan-PI3K inhibitors at 0.1 or 1 µM and concomitantly subjected to a Boyden chamber migration assay. Migrating cells were quantified after 24 h. **D**, Migration inhibition capacities of PI3K inhibitors at 1 µM were tested on HPNE hTERT cells and were plotted against the in vitro IC50 for each class I PI3K of each inhibitor determined on recombinant proteins. Pearson correlation tests were performed. Mean +/- SEM (* p<0.05, ** p<0.01, *** p<0.001, n≥3, Student's t-test).

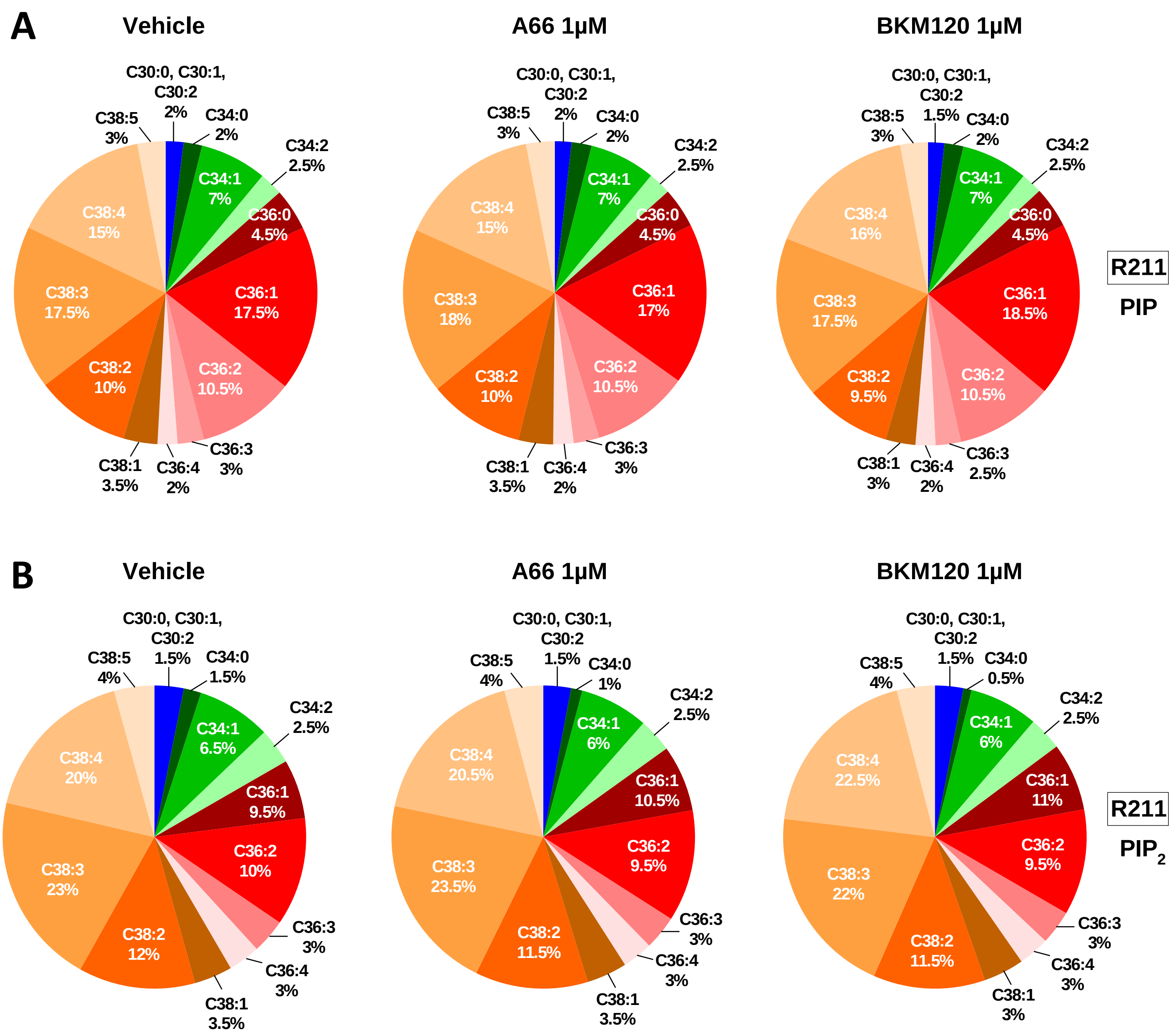

Appendix Figure S8: PIP and PIP_2 sub-types. Murine pancreatic tumour cells R211 are treated for 15 min with the vehicle (0.01% DMSO) or α-specific A66 or pan-PI3K inhibitor BKM120 at 1µM. Phospholipids were extracted and the proportion of each **A**, PIP and **B**, PIP2 subtype was represented. N=3

A

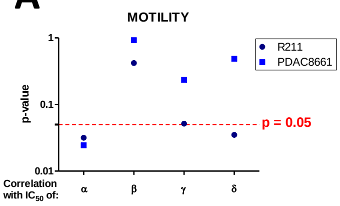

B

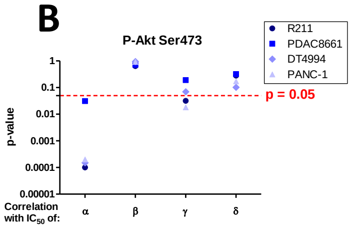

C

		Migration correlation - R squared			
		PI3Kα	PI3Kβ	PI3Kγ	PI3Kδ
Pancreas	R211	0.6707	0.01982	0.2436	0.1686
	PDAC8661	0.7075	0.0005708	0.3281	0.2799
	PANC-1	0.5336	0.0397	0.1811	0.3739
	10593	0.8969	0.6883	0.04761	0.8156
	10158	0.792	0.3554	0.354	0.5543
	R6344	0.388	0.06159	0.1963	0.3665
	R6430	0.6669	0.001815	0.3333	0.2532
	R6065	0.6097	0.2529	0.2354	0.6925
	R6141	0.5217	0.1696	0.2579	0.6677
Other organs	PC3	0.06767	0.8464	0.07215	0.7513
	MDA-MB-231	0.1328	0.1938	0.1641	0.3925
	MDA-MB-468	0.05677	0.4916	0.05836	0.5726

D

		Cytotoxicity correlation - R squared			
		PI3Kα	PI3Kβ	PI3Kγ	PI3Kδ
Pancreas	R211	0.3917	0.01859	0.2506	0.2026
	PDAC8661	0.5084	0.01331	0.3061	0.2586
	DT4994	0.5554	0.02876	0.3779	0.2945
	PANC-1	0.3131	0.1128	0.2374	0.3323
	10593	0.7159	0.6617	0.113	0.7343
	10158	0.7154	0.527	0.2357	0.6636
	R6344	0.7754	0.01008	0.3046	0.2667
	R6430	0.6886	0.007813	0.238	0.2871
	R6065	0.7842	0.005775	0.3083	0.2899
	R6141	0.6351	0.0555	0.3203	0.4164
Other organs	PC3	0.3739	0.3731	0.2671	0.6739
	MDA-MB-231	0.2255	0.07722	0.2499	0.2245
	MDA-MB-468	0.2327	0.2938	0.2018	0.4658
	NOMO-1	0.5756	0.006474	0.5803	0.169
	HL-60	0.4795	0.001741	0.7097	0.09689

E

		Motility correlation - R squared			
		PI3Kα	PI3Kβ	PI3Kγ	PI3Kδ
Pancreas	R211	0.8291	0.2247	0.7671	0.8178
	PDAC8661	0.8556	0.003624	0.4237	0.174

F

		p-Akt Ser473 correlation - R squared			
		PI3Kα	PI3Kβ	PI3Kγ	PI3Kδ
Pancreas	R211	0.8288	0.02511	0.4175	0.1272
	PDAC8661	0.4189	0.01634	0.1822	0.109
	DT4994	0.8141	0.0004657	0.3192	0.2686
	PANC-1	0.8671	0.001017	0.4797	0.1964

G

		R211	DT4994	A260	10158	A338	PDAC8661	R6065	A94L	10593	R6430	R6141	A338L	R6344
Epithelial	E-cadherin	0.00	-0.39	-0.42	-0.19	-0.62	-0.30	0.30	-0.39	-0.17	-0.49	-0.74	-4.91	-4.08
	CK19	0.00	-1.15	-0.66	-0.75	-1.08	-0.58	-0.39	-0.71	-0.33	-0.64	-0.93	-3.26	-3.80
	CK7	0.00	-0.52	-1.20	-2.25	-2.83	-0.18	0.12	0.13	-1.81	-1.44	-1.36	-1.93	-3.26
	Collagen IV alpha 1	0.00	-0.20	-0.30	-0.59	-0.22	-0.24	-0.06	-0.36	0.32	0.04	-0.07	-0.11	0.28
	Desmoglein-3	0.00	0.35	0.05	0.42	-0.17	-0.92	-0.33	-0.78	0.84	-0.39	0.36	0.21	-1.17
	MUC-1	0.00	-1.28	-1.46	-1.65	-1.60	-0.70	-1.03	-0.96	-0.72	-1.73	-1.85	-2.31	-2.92
	Syndecan-1	0.00	-0.56	-0.15	-0.65	-0.70	-0.29	0.19	-0.24	-0.06	-0.45	-0.08	-1.33	-0.72
Mesenchymal	S100A4	0.00	0.01	-1.61	-0.97	-0.30	-0.37	-1.22	-0.69	-0.46	-0.34	-0.59	0.25	0.52
	FN1	0.00	-0.80	-0.68	-1.36	-0.66	-0.55	-0.20	-1.59	-0.60	-0.54	0.34	-0.47	-0.07
	Snail	0.00	-1.13	-1.73	-0.92	-1.07	-1.00	-0.46	-1.18	0.51	-0.23	-0.12	0.60	0.49
	Twist	0.00	-0.19	-0.94	-0.79	-1.69	-0.33	-0.26	0.36	-0.13	-0.13	0.30	0.16	0.14
	Col1A1	0.00	-2.23	-2.26	-1.54	-0.92	-1.43	-1.30	0.27	1.88	1.99	-0.85	2.16	1.85
	Alpha-SMA (Acta2)	0.00	-2.78	-3.18	-1.84	-1.85	-1.08	-0.19	-0.51	0.63	1.85	1.77	2.16	1.97
	Vimentin	0.00	-0.52	-0.18	-0.59	-0.34	-0.31	-0.16	-0.44	0.00	-0.27	-0.16	-0.26	-0.47
Subtype		Epithelial								Mesenchymal				

Relative expression (Log 10)
-5.00
-4.00
-3.00
-2.00
-1.00
0.00
1.00
2.00
3.00
4.00
5.00

H

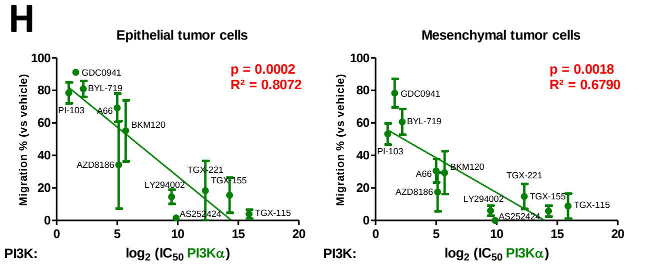

I

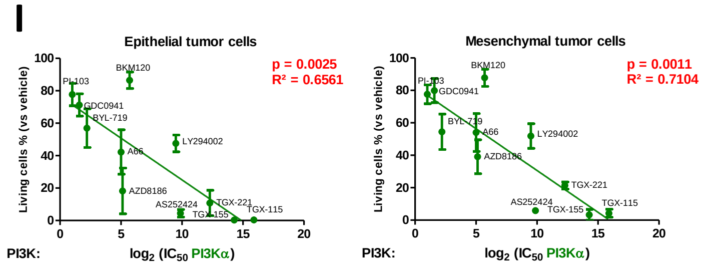

Appendix Figure S9: Correlations between cellular phenotypes and class I PI3K isoform inhibition. Inhibitory capacities of PI3K inhibitors at 1 µM on motility and at 10 µM on Akt phosphorylation (Ser473) were plotted against the in vitro IC50 for each class I PI3K (α, β, γ and δ) of each inhibitor determined on recombinant proteins. Pearson correlation tests were performed. The p-value obtained was represented for each tested cell line and for each class I PI3K isoform for **A**, motility and **B**, Akt phosphorylation on Ser473. The dotted red line corresponds to a p-value of 0.05. Individual R squared values of the Pearson correlation test for each cell line (from the pancreas or other organs) and each PI3K isoform are shown for **C**, migration, **D**, cytotoxicity, **E**, motility and **F**, Akt phosphorylation on Ser473. **G**, The expression of epithelial and mesenchymal markers was assessed by RT-qPCR in all pancreatic murine tumour cells and represented as log10 of fold expression, with R211 being the reference cell line. **H**, Migration inhibition or **I**, cytotoxic capacities of PI3K inhibitors at 1 and 10 µM respectively were tested on epithelial or mesenchymal pancreatic murine tumour cells. The mean of these values was plotted against the in vitro IC50 for PI3Kα and each inhibitor. IC50 is determined on recombinant proteins. Pearson correlation tests were performed and p-values were presented. Mean +/- SEM (* $p<0.05$, ** $p<0.01$, *** $p<0.001$, $n\geq3$, Student's t-test).

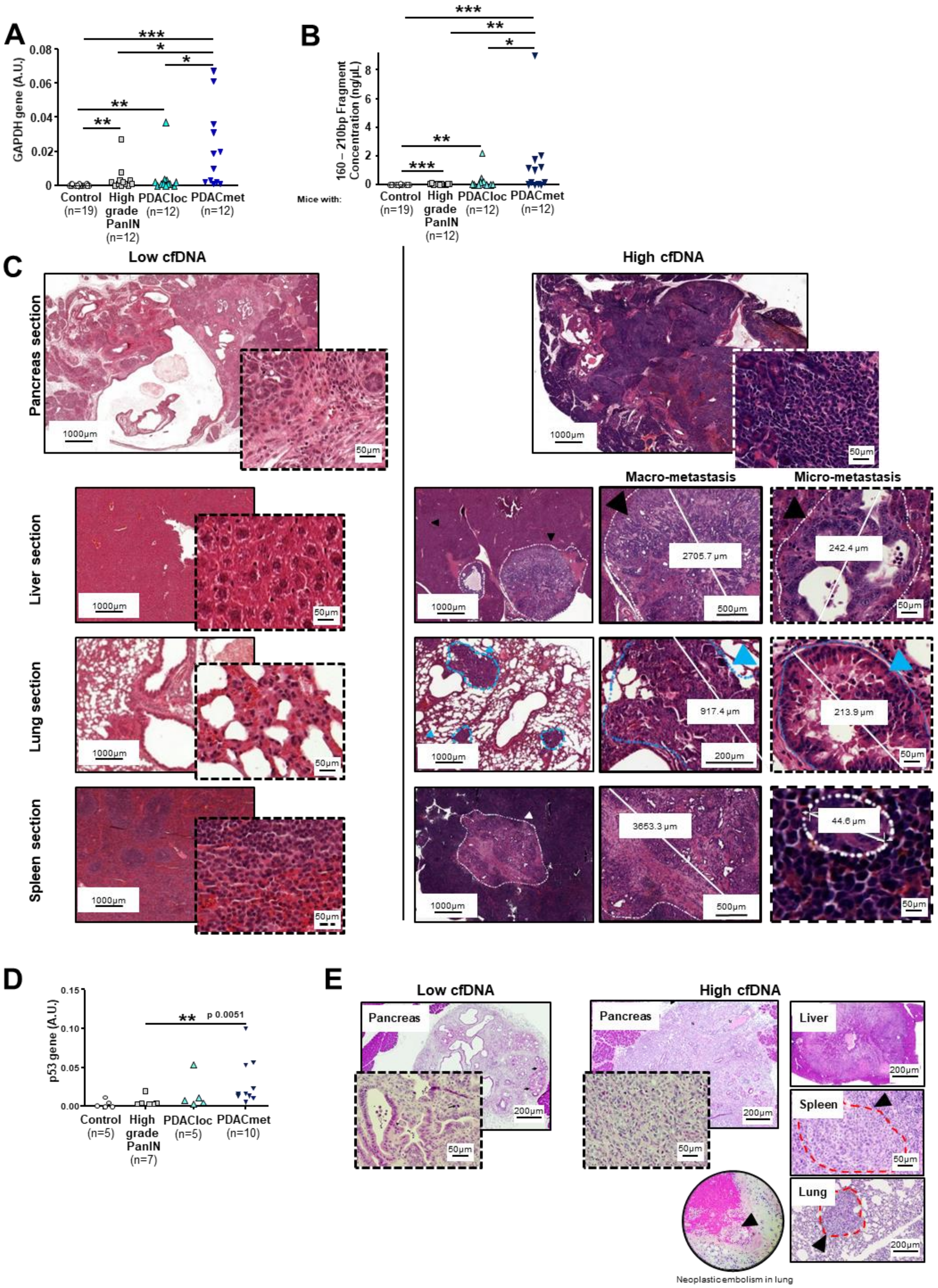
A
GAPDH gene (A.U.)
Control (n=19)
High grade PanIN (n=12)
PDACloc (n=12)
PDACmet (n=12)
B
160 – 210bp Fragment Concentration (ng/µL)
Mice with:
C
Low cfDNA
High cfDNA
Pancreas section
Liver section
Lung section
Spleen section
Macro-metastasis
Micro-metastasis
1000µm
50µm
500µm
200µm
2705.7 µm
242.4 µm
917.4 µm
213.9 µm
3653.3 µm
44.6 µm
D
p53 gene (A.U.)
p 0.0051
Control (n=5)
High grade PanIN (n=7)
PDACloc (n=5)
PDACmet (n=10)
E
Low cfDNA
High cfDNA
Pancreas
Liver
Spleen
Lung
200µm
50µm
Neoplastic embolism in lung

Appendix Figure S10: Additional information on cfDNA in KPC mice. A, Quantification of cfDNA by GAPHD gene in KPC mice at different stages of the disease. **B,** Quantification of 160-210bp Fragment concentration in KPC mice at different stages of the disease. N in each group is indicated. **C**, Representative images of KPC mice with low and high levels of cfDNA. **D**, Quantification of cfDNA and **E,** representative images of KPC mice originating from another animal house (CRCM Marseille). N is indicated.

A

Enrolment criteria for KPC mice	
Detected tumour by US imaging	
Tumour location	Head or tail
Increased cfDNA*	
Weight	> 22g
Sex	Male or female

B

Mouse ID	Sex	Age (weeks)	Initial weight (g)	cfDNA (Fold increase)	Tumour location	Treatment
637	M	35	25.6	2.333	Head	BYL-719
715	M	32	27.3	4.000	Head	BYL-719
773	F	29	25.2	1.000	Head	BYL-719
785	M	30	32.5	0.500	Tail	BYL-719
871	M	24	36.3	0.750	Tail	BYL-719
897	M	21	36.1	5.750	Head	BYL-719
790	M	32	28	1.500	Tail	Vehicle
795	F	33	24.7	1.667	Head	Vehicle
860	M	23	30.4	0.222	Head	Vehicle
894	M	25	32	3.500	Tail	Vehicle
939	F	18	22.3	2.167	Head	Vehicle
977	M	20	30.9	6.333	Tail	Vehicle

Appendix Figure S11: Description of the KPC cohort. A, Enrolment criteria for KPC mice included in the study. **B,** Detailed description of KPC study mice.

A

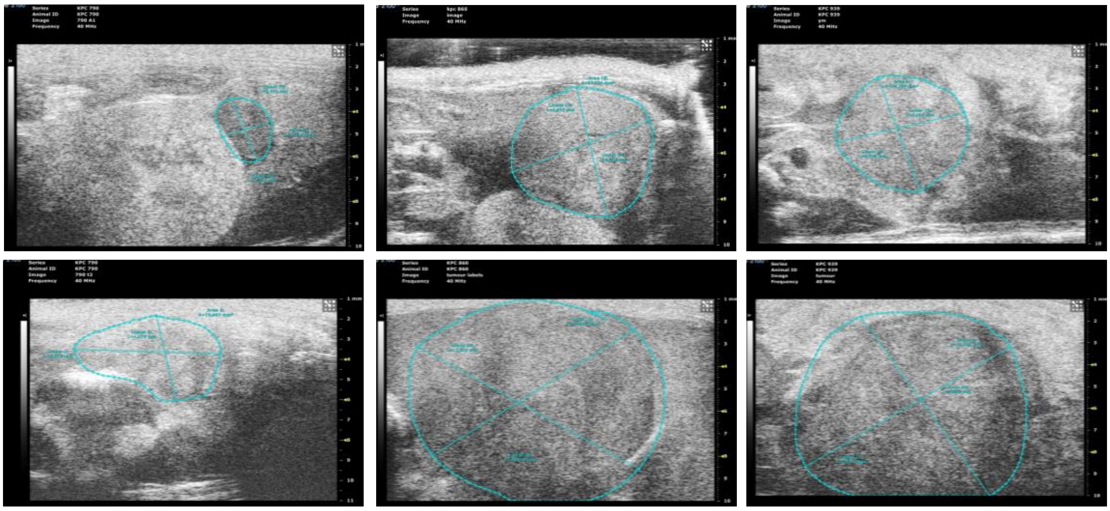

B

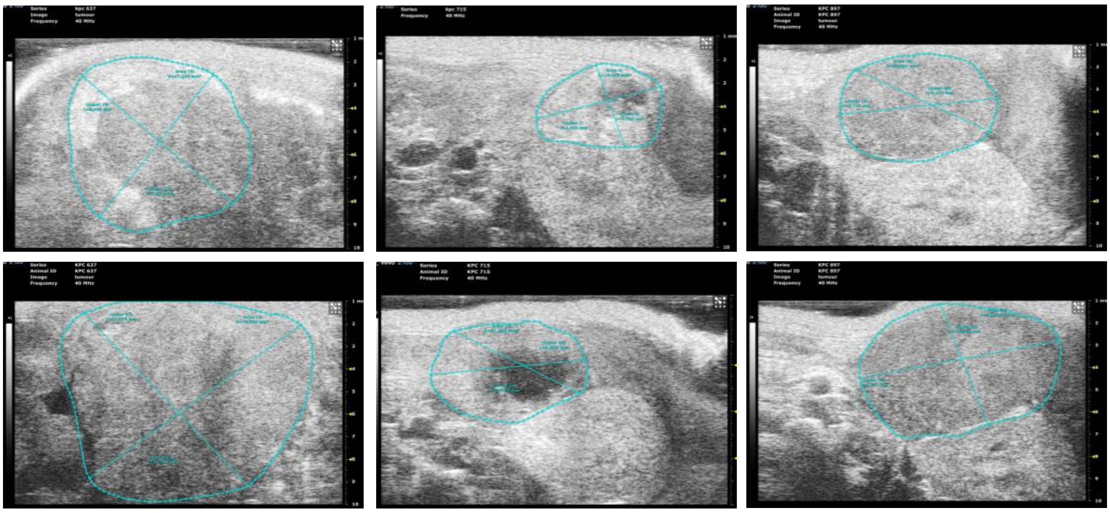

C

Mouse ID	Initial Tumour volume (mm^3)	Final Tumour volume (mm^3)	Tumour fold change	Treatment
637	435.523	656.492	1.507	BYL-719
715	62.045	97.522	1.572	BYL-719
773	156.436	555.745	3.553	BYL-719
785	256.423	403.214	1.572	BYL-719
871	288.199	357.401	1.240	BYL-719
897	181.604	265.693	1.463	BYL-719
790	14.671	154.878	10.557	Vehicle
795	450.402	821.260	1.823	Vehicle
860	465.018	1045.508	2.248	Vehicle
894	179.036	458.345	2.560	Vehicle
939	93.553	620.745	6.635	Vehicle
977	435.572	1041.951	2.392	Vehicle

Appendix Figure S12: Tumour progression by US imaging. Representative US images of tumour progression in KPC mice treated with **A,** Vehicle and with **B,** BYL-719. **C,** Summary of tumour progression in the treated KPC mice.

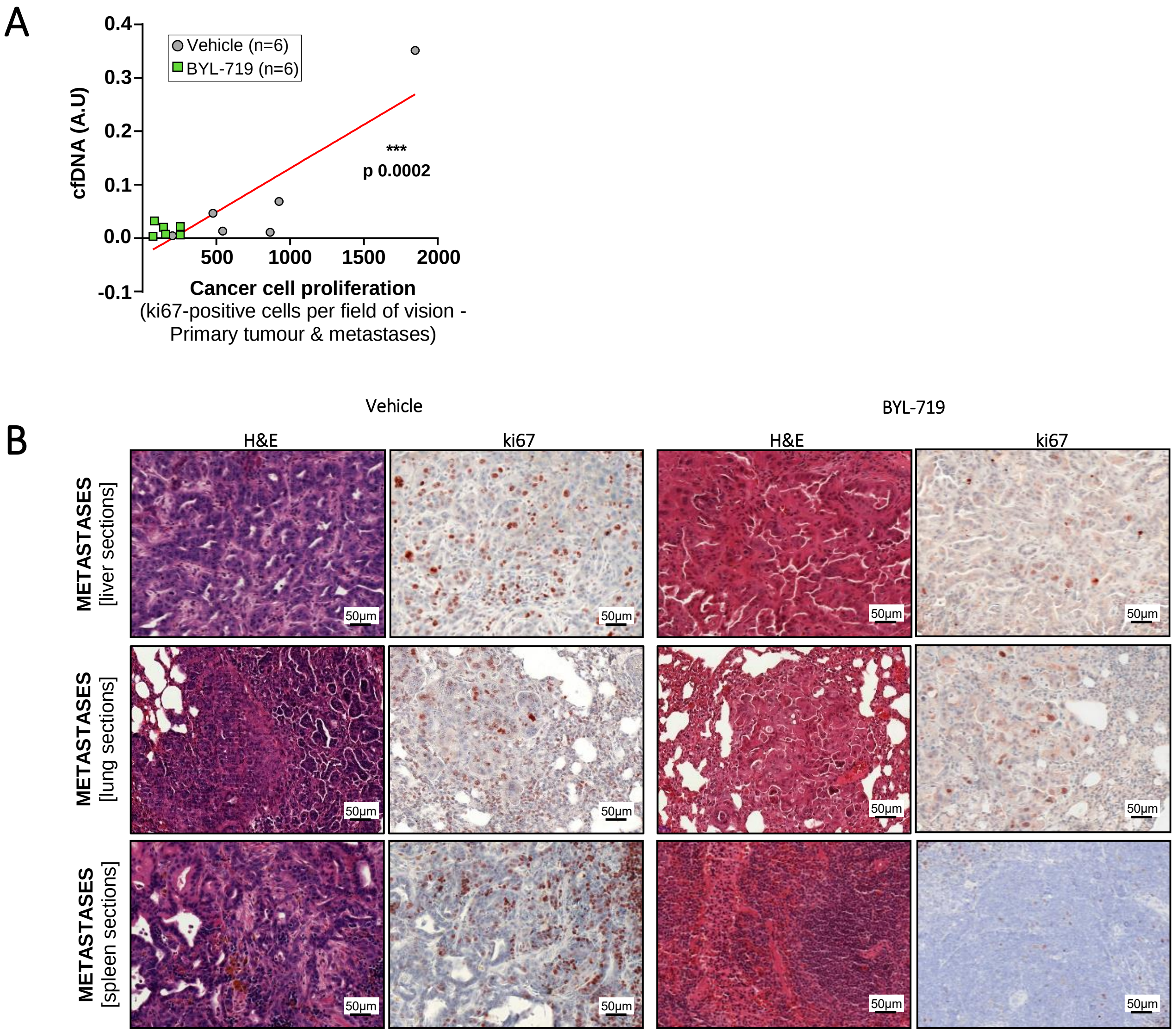

Appendix Figure S13: Effects of PI3Kα pharmacological inhibition on cfDNA and metastasis. A, Significant correlation between cfDNA and cancer cell proliferation in treated KPC mice. **B,** Representative images of (H&E and Ki67 IHC) metastases in liver, lung and spleen of KPC mice treated with the vehicle or BYL-719. N is indicated. Mean +/- SEM (* p<0.05, ** p<0.005, *** p<0.0001).

A

Mouse ID	Treatment	Tumour location	Primary tumour	Histological Grade	Regional lymph nodes	Distant Metastases	Metastasic sites	Stage
B-637	BYL-719	Head	T4	G3	N1	M1	Liver, lung, peritoneum,	IV
B-715	BYL-719	Head	T2	G2	N0	M0	N/A	IB
B-773	BYL-719	Head	T4	G3	N1	M1	Liver, peritoneum, ascites	IV
B-785	BYL-719	Tail	T4	G3	N0	M0	N/A	III
B-871	BYL-719	Tail	T3	G3	N0	M0	N/A	III
B-897	BYL-719	Head	T4	G3	N1	M1	Liver	IV
V-790	Vehicle	Tail	T2	G4	N0	M0	N/A	IB
V-795	Vehicle	Head	T4	G3	N1	M1	Liver, lung, spleen, peritoneum, ascites	IV
V-860	Vehicle	Head	T4	G3	N1	M1	Liver, spleen, peritoneum, ascites	IV
V-894	Vehicle	Tail	T4	G4	N1	M1	Liver, spleen, peritoneum, ascites	IV
V-939	Vehicle	Head	T4	G4	N1	M1	Liver, lung, peritoneum, ascites	IV
V-977	Vehicle	Tail	T4	G4	NX	M1	Liver, peritoneum	IV

Primary Tumour (T)
TX Primary tumour cannot be assessed
T0 No evidence of primary tumour
Tis Carcinoma in situ* includes PanIN III
T1 Tumour limited to the pancreas, less than 2mm in biggest dimension
T2 Tumour limited to the pancreas, more than 2mm in biggest dimension
T3 Tumour extends beyond the pancreas but without involvement of the celiac axis or main arteries
T4 Tumour involves celiac axis or main arteries (unresectable primary tumour)

Regional Lymph Nodes (N)
NX Regional lymph nodes cannot be assessed
N0 No regional lymph node metastasis
N1 Regional lymph node metastasis

Distant Metastases (M)
M0 No distant metastases
M1 Distant metastases

Histological grade (G)
GX cannot be assessed
G1 well differentiated
G2 moderately differentiated
G3 poorly differentiated
G4 undifferentiated

B

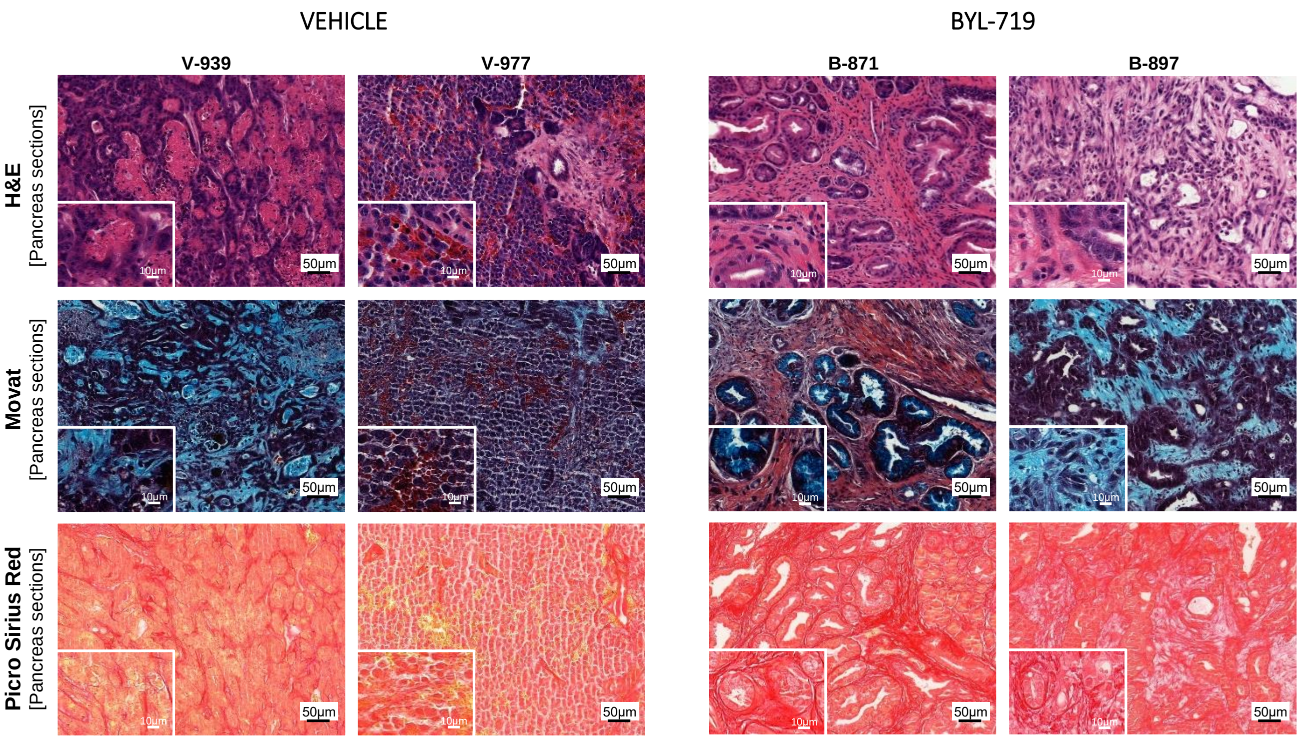

Appendix Figure S14: Cancer staging and tumour histology of KPC mice treated with the vehicle and BYL-719. A, Cancer staging of KPC mice treated with the vehicle and BYL-719. **B,** Representative images of tumours in KPC mice. H&E, Movat's Pentachrome and Picro Sirius Red staining.

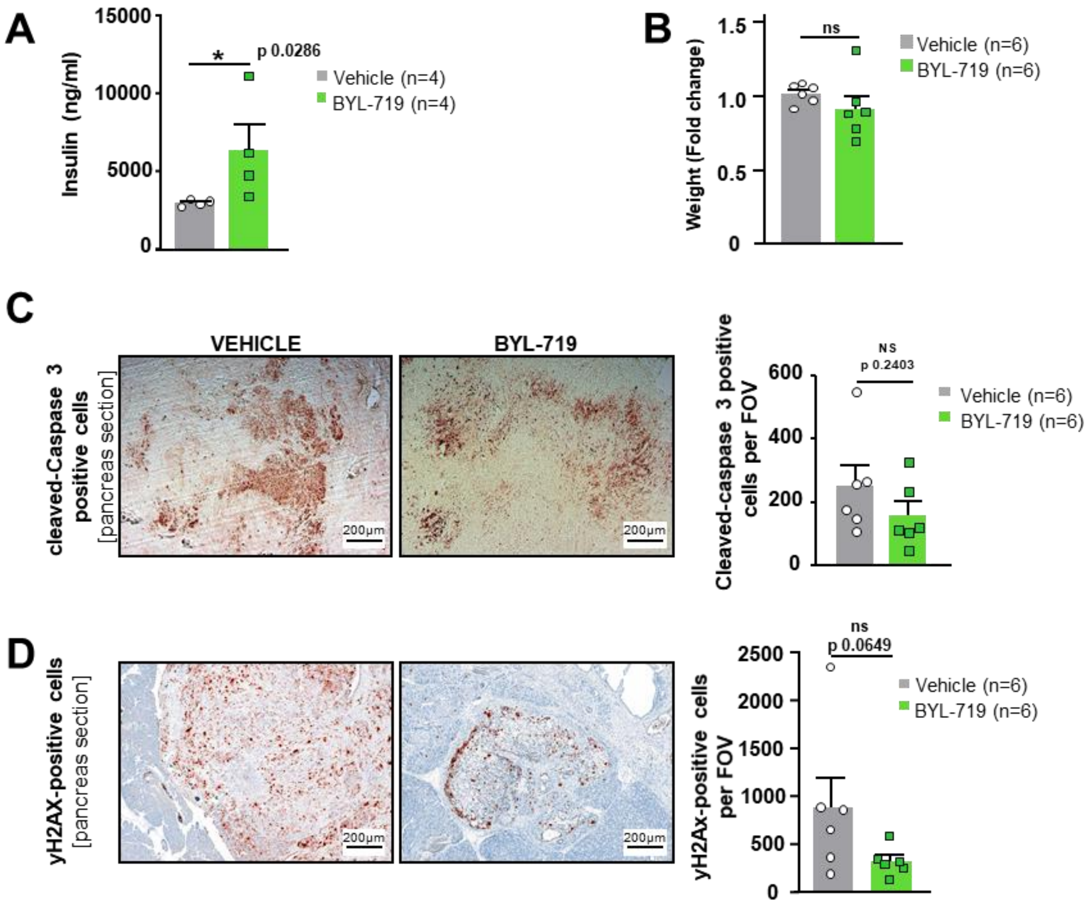

Appendix Figure S15: Secondary effects of PI3Kα pharmacological inhibition. A, Insulin quantification on treated KPC. **B,** Quantification of weight change in treated KPC mice. **C,** Representative images and quantification of cleaved Caspase 3 and **D,** γH2AX –positive cells in pancreatic sections of treated KPC mice. Mean +/- SEM (* p<0.05, ** p<0.005, *** p<0.0001)

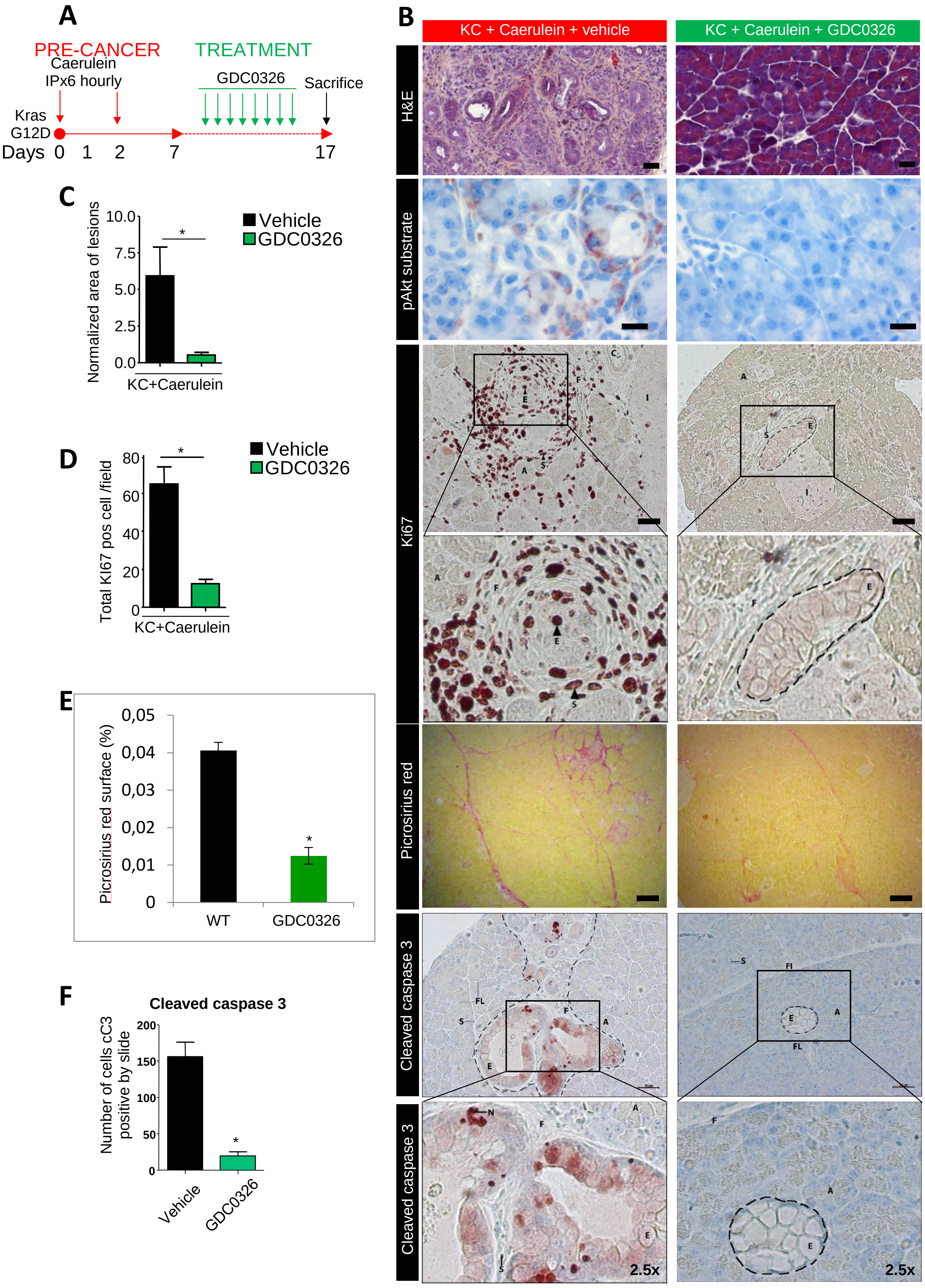
A
PRE-CANCER
TREATMENT
Caerulein
IPx6 hourly
GDC0326
Sacrifice
Kras
G12D
Days 0 1 2 7 17
B
KC + Caerulein + vehicle
KC + Caerulein + GDC0326
H&E
pAkt substrate
Ki67
Picrosirius red
Cleaved caspase 3
Cleaved caspase 3
2.5x
2.5x
C
Normalized area of lesions
10.0
7.5
5.0
2.5
0.0
*
Vehicle
GDC0326
KC+Caerulein
D
Total Ki67 pos cell /field
80
60
40
20
0
*
Vehicle
GDC0326
KC+Caerulein
E
Picrosirius red surface (%)
0,05
0,04
0,03
0,02
0,01
0
*
WT
GDC0326
F
Cleaved caspase 3
Number of cells cC3 positive by slide
200
150
100
50
0
*
Vehicle
GDC0326

Appendix Figure S16: Pharmacological inactivation of PI3Kα reverses pre-cancer lesions and alterations of their stromal environment in an oncogenic KRAS context. A. Dosage regimen of PI3Kα-targeting drug (GDC0326 10mg/kg daily- blue arrows) in KC mice subjected to acute pancreatitis protocol with caerulein as described in Baer et al (black arrows). **B-F** IHC, stainings and quantification as indicated after 17 days; n=4 mice in each group. The surface of the lesion areas was quantified on two whole slides for each mouse, and normalised with the total area of the pancreas. Values are mean ± SEM. T-Test: *=P<0.05 Representative images are shown. A: acinar cells, E: epithelial lesion; FL=fibroblasts, S: stroma, I: Langerhans islet

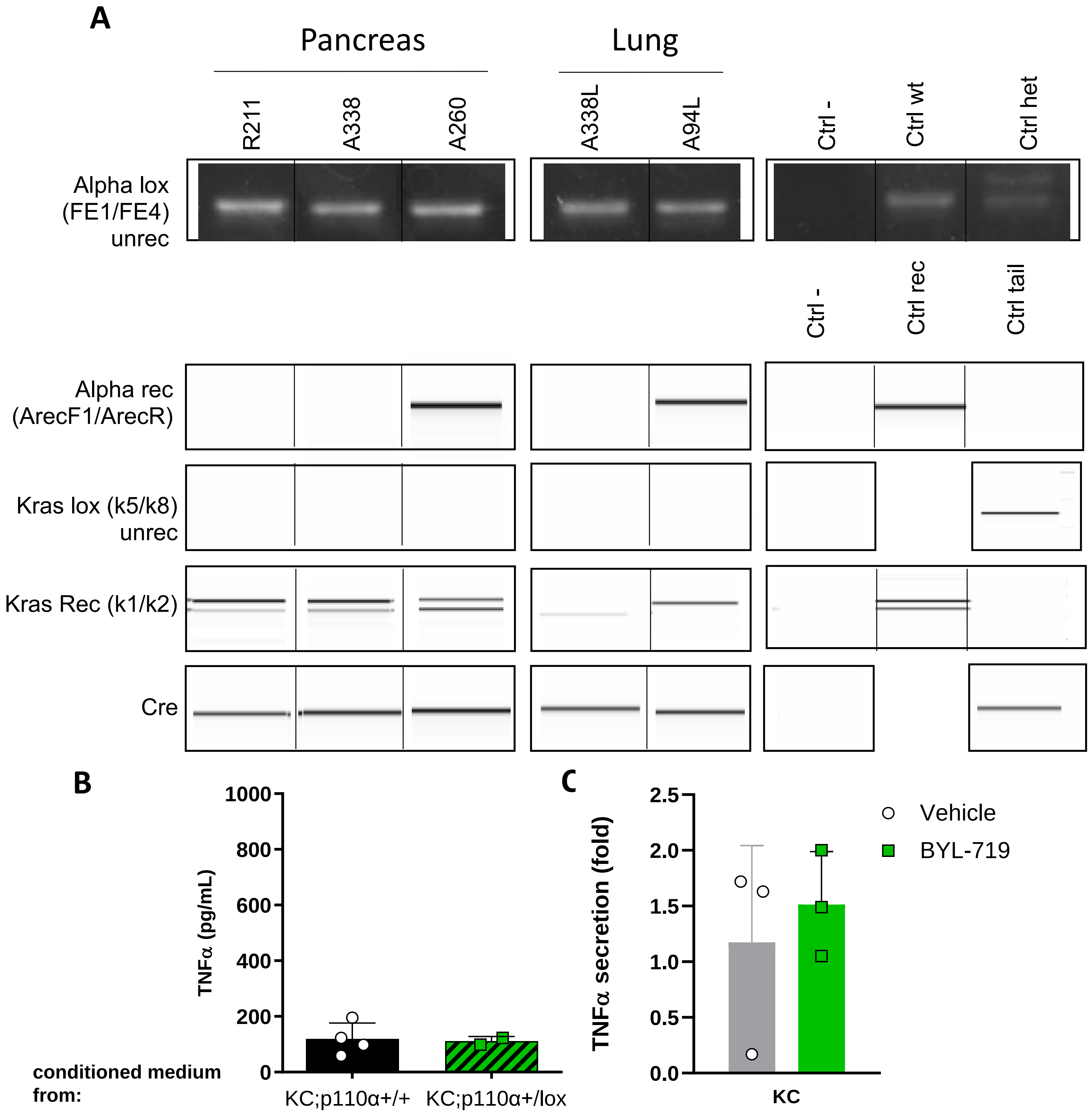

Appendix Figure S17: Murine cell lines derived for the study. A, Genotyping. **B**, Quantification of secreted TNFα by tumour cells as indicated.

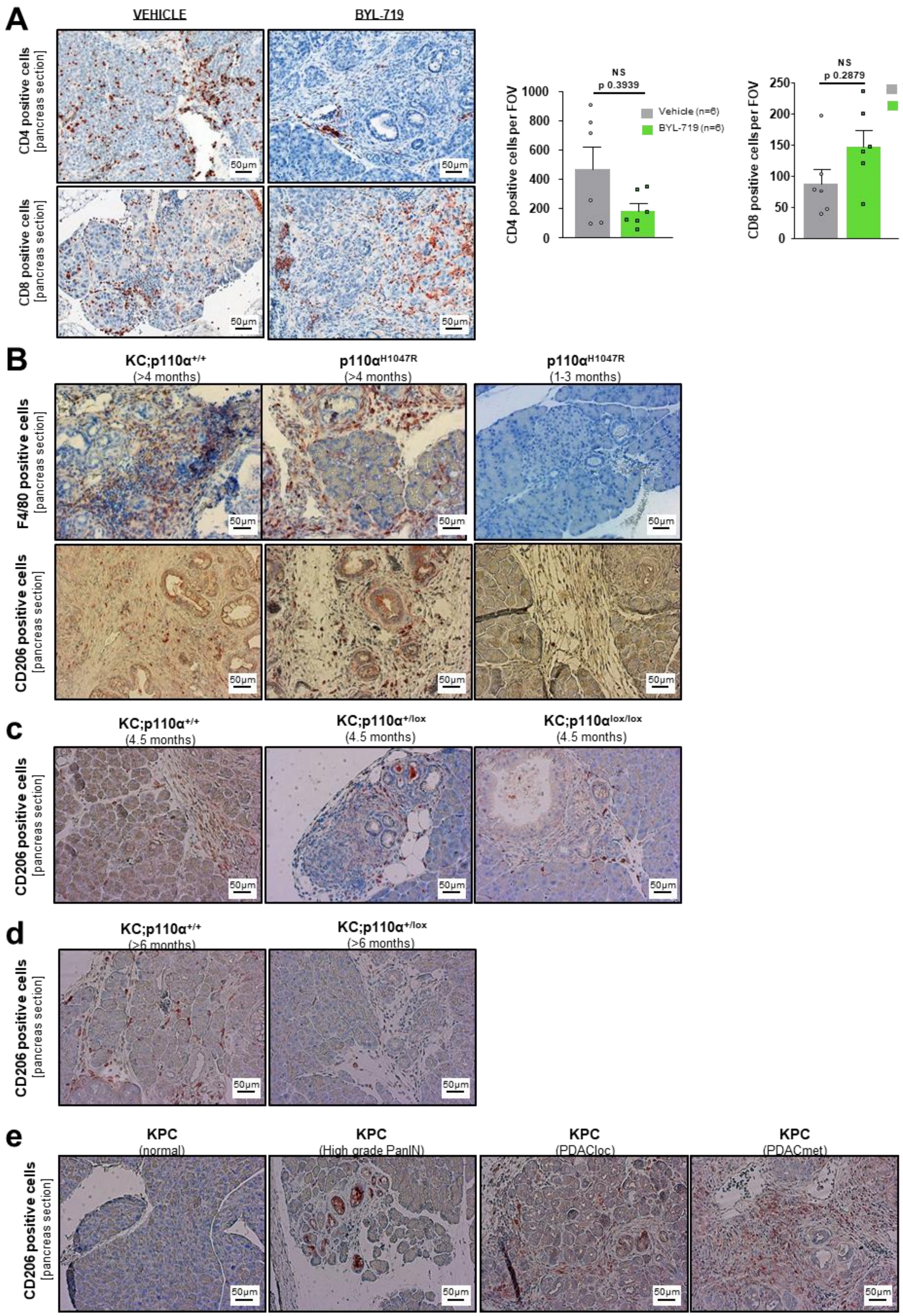

A
VEHICLE
BYL-719
CD4 positive cells [pancreas section]
CD8 positive cells [pancreas section]
50µm
NS
p 0.3939
Vehicle (n=6)
BYL-719 (n=6)
CD4 positive cells per FOV
0
200
400
600
800
1000
NS
p 0.2879
CD8 positive cells per FOV
0
50
100
150
200
250
B
KC;p110α+/+ (>4 months)
p110αH1047R (>4 months)
p110αH1047R (1-3 months)
F4/80 positive cells [pancreas section]
CD206 positive cells [pancreas section]
c
KC;p110α+/+ (4.5 months)
KC;p110α+/lox (4.5 months)
KC;p110αlox/lox (4.5 months)
CD206 positive cells [pancreas section]
d
KC;p110α+/+ (>6 months)
KC;p110α+/lox (>6 months)
CD206 positive cells [pancreas section]
e
KPC (normal)
KPC (High grade PanIN)
KPC (PDACloc)
KPC (PDACmet)
CD206 positive cells [pancreas section]

Appendix Figure S18: Effects of PI3Kα inhibition on the immune response. **A,** Representative images and quantification of CD4 and CD8 positive cells in KPC mice treated with the vehicle or BYL-719. N is indicated. **B,** Representative images of F4/80 and CD206 positive cells in KC and oncogenic PI3Kα mice at different ages. **C,** Representative images of CD206 positive cells around low-grade PanIN lesions in KC; p110α$^{+/+}$, KC; p110α$^{+/lox}$ and KC; p110α$^{lox/lox}$ mice (4.5 months). **D,** Representative images of CD206 positive cells around high-grade PanIN lesions in KC; p110α$^{+/+}$ and KC; p110α$^{+/lox}$ (>6 months). **E,** Representative images of CD206 positive cells in KPC mice with normal, high-grade PanIN, PDACloc and PDACmet pancreas.

Appendix Additional methods

Detailed genotyping of mice

The compound mutants LSL-Kras^{G12D}-LSL-p53^{R172H}-Pdx1-Cre (KPC), LSL-KRAS^{G12D}-Pdx1-Cre (KC), LSL-KRAS^{G12D}-Pdx1-Cre-p110α$^{+/lox}$ (KC;p110α$^{+/lox}$), KRAS^{G12D}-Pdx1-Cre-p110α$^{+/lox}$ (KC; p110α$^{lox/lox}$), LSL-KRAS^{G12D}-LSL-p53^{R172H}-Pdx1-Cre- p110α$^{+/lox}$ (KPC; p110α$^{+/lox}$), LSL-KRAS^{G12D}-LSL-p53^{R172H}-Pdx1-Cre-p110α$^{+/lox}$ (KPC; p110α$^{lox/lox}$) were obtained through breeding. Littermates not expressing Cre were used as controls. Genotyping was performed as described in (Baer *et al*, 2014; Hingorani *et al*, 2005; Therville *et al*, 2019) and analysed with the Fragment Analyzer Systems (AATI) DNA Analysis Kits dsDNA 910 Reagent kit, 35-1500bp (AATI). The genotyping of p53 allele uses T035- 5'-CTTGGAGACATAGCCACACTACTG-3'/ T036 - 5'-AGCTAGCCACCATGGCTTGAGTAAGTCTGCA-3' / T037 - 5'-TTACACATCCAGCCTCTGTGG-3'- Annealing temperature: 60°C – amplicon size for WT: 166bp, for LSL-p53R172H: 270bp. Animal sample size was calculated using a power analysis in order to obtain statistically significant results from ki67 (n=5, p≤0.02) and CD206 (n=6, p≤0.05) immune staining (Table EV5).

Detailed protocol for primary murine cancer cell isolation and culture

Organs were dissected using a scalpel in DMEM containing 200 U/ml of collagenase (Sigma C6885), incubated for 20 minutes in a water bath at 37°c under agitation. After washing, the cell suspension was resuspended in 10% DMEM FBS, filtered through a 100 µm sieve and seeded in a 10-cm cell dish. After 7 passages, genotyping was carried out (Appendix Figure S17) with confirmed recombination of KRAS (PCR for detection of unrec (positive control: R211 cell line). LSL-KRASG12D allele: K005 5'-AGCTAGCCACCATGGCTTGAGTAAGTCTGCA-3' / K008 5'-TCCGAATTCAGTGACTACAGATGTACAGAG-3' – annealing temperature: 55°C - size of amplicon: 300bp; PCR for detection of recombined LSL-KRASG12D: K001 5'-GTCTTTCCCCAGCACAGTGC-3' / K002: 5'-CTCTTGCCTACGCCACCAGCTC-3' - annealing temperature: 61°C - size of amplicon: doublet at 554bp) without (R211, A338, A338L cell lines) or with *Pik3ca* gene recombination (A260, A94L cell lines) (PCR for detection of loxed *Pik3ca*

gene: FE1-5’-GGATGCGGTCTTTATTGTC-3’ / FE4- 5’-TGGCATGCTGCCGAATTG-3’ - annealing temperature: 59°C - amplicon size for WT: 640bp, for unrec. lox : 708 bp; PCR for detection of rec *Pik3ca* gene : aRecF1 5’-GGGGACAGTAGGAGGATGGT-3’ / aRecR 5’-TGGCATTCCAGAGCCAAGC-3’ - annealing temperature: 65°C - amplicon size : 812bp); it should be noted that recombination is driven by Cre expression in pancreatic lineage (Pdx1-cre: H06: 5’-TGCCACGACCAAGTGACAGCAATG-3’; H07 5’-GACCAGAGTCATCCTTAGCGCCG-3’ – annealing temperature: 65°C - amplicon size: 580bp). Source data of cell genotyping is available. Tumorigenicity of A338 and A260 cell lines was verified by injection ($7.5x10^4$ or $1.25x10^5$ cells) in Nude and NSG mice. Cells were passaged several times before experiments to avoid stromal cell contamination.

Detailed Cell culture

All cells were cultured in DMEM (Dulbecco's Modified Eagle Medium), 4.5 g of glucose supplemented with 10% foetal bovine serum (FBS), 1% L-glutamine, 1% penicillin/streptomycin and 0.01% plasmocin, except for HL-60, NOMO-1 and PC3 cultured in RPMI (Roswell Park Memorial Institute) supplemented as described previously or HPNE hTERT that were cultured in 75% DMEM without glucose/25% medium M3 Base supplemented with 5% FBS, 10 ng/ml human recombinant EGF, 5.5 mM D-glucose (1g/L) and 750 ng/ml puromycin or HPDE cells cultured in keratinocyte serum-free (KSF) medium supplemented by epidermal growth factor and bovine pituitary extract. Cells were cultured at 37°C in a humidified 5% CO_2 atmosphere. AML: Acute myeloid leukaemia; WT: Wild type. Cells were checked monthly by PCR for mycoplasma infection using the following primers: Forward: GCTGTGTGCCTAATACATGCAT and Reverse: ACCATCTGTCACTCTGTTAACCTC. Genomic DNA from a mycoplasma-infected cell line was used as a positive control.

Detailed methods in cell assays

Cytotoxicity assay

Cells were seeded in 96-well plates. Twenty-four hours later, they were treated with a dose range of α-selective, β-selective, β/δ-selective, γ-selective or pan PI3K inhibitors at 0.1, 1 and 10 µM,

respectively, in 10% FBS medium or the vehicle (DMSO 0.1%). For IC50 value, see Table EV4B. Three day post-dose, living cells were stained using MTT for adherent cell lines or MTS for acute myeloid cell lines. Control cells were treated with 0.1% DMSO and considered as 100% living cells. Each condition was conducted in triplicate and each experiment was performed at least three times.

Apoptosis

Cells ($5x10^3$) were seeded in 96-well plates. Twenty-four hours after seeding, cells were treated with α-selective, β-selective, β/δ-selective, γ-selective or pan PI3K inhibitors at 1 and 10 µM, in 10% FBS medium or the vehicle (DMSO 0.1%) and were marked for apoptotic cells with IncuCyte® Annexin V (red) according to the manufacturer’s protocols (Sartorius). Confluence and fluorescence were monitored every 2 h with IncuCyte S3 (Sartorius). Control cells were treated with 0.1% DMSO. Apoptotic cell surface was normalized with confluence and the fold change was calculated by comparing the treatments to the vehicle condition. Each condition was conducted in triplicate and each experiment was performed three times.

Motility assay

Cells ($1x10^5$) were seeded in 24-well plates. Scratching was carried out at confluence using a pipette tip and cells were treated with α-selective and pan-PI3K inhibitors at 0.01, 0.1 or 1 µM, or the vehicle (0.01% DMSO). Cells were examined microscopically after 8 h and 24 h, and the scratch surface was analysed using ImageJ software. Control cells were treated with 0.01% DMSO. Five photographs were taken per condition and each experiment was carried out at least three times.

Migration assay

Cells ($5x10^4$) were seeded in the upper compartment of Boyden chamber inserts in serum-free DMEM. Four hours after seeding, complete DMEM was added to the lower compartment to trigger migration. Cells were treated with α-selective, β-selective , β/δ-selective , γ-selective and pan-PI3K inhibitors at 0.1 and 1 µM, an FGFR inhibitor (AZD4547) at 2 µM, TNF-α at 20 ng/mL or the vehicle (0.01% DMSO) in the lower and upper compartments. Twenty-four hours after treatment, cells were fixed with 3.7% formaldehyde for 20 minutes and then stained with crystal violet for 20 minutes. A

cotton swab was used to remove non-migrated cells from the insert. Migrated cells were analysed using ImageJ software. Control cells were treated with 0.01% DMSO. Ten photographs were taken per condition and each experiment was carried out at least three times.

Western blot

Cells were scraped in cold lysis buffer (50mM Tris at pH 7.4, 150 mM NaCl, 1% Triton, 1 mM EDTA, 2 mM DTT, 2 mM NaF, 4 mM Na orthovanadate and a protease inhibitor cocktail provided by Roche). Fifty micrograms of proteins were separated by SDS-PAGE and transferred to a PVDF membrane. Membranes were saturated in TBS (50 mM Tris, 150 mM NaCl)/0.1% Tween 20 (TBST)/5% milk and incubated with the primary antibody (Table EV4C). Membranes were washed with TBST and incubated with the corresponding secondary antibody. Membranes were washed with TBST and immunocomplexes were visualised using ECL RevelBlot Plus (Ozyme) by autoradiography.

RNA extraction and RT-qPCR

The cell pellet was resuspended in Trizol (Invitrogen). Chloroform was added and the suspension was centrifuged for 10 minutes at 18,000 g at 4°C. The aqueous phase was isolated, completed with isopropanol then centrifuged for 10 minutes at 18,000 g at 4°C. The pellet was washed with cold 80%, 83% EtOH. The suspension was centrifuged for 5 minutes at 18,000 g at 4°C and the pellet was dried and then resuspended in RNAse-free water. 1 µg of extracted RNA was used to obtain cDNA using RevertAid H minus reverse transcriptase, random hexamers and the corresponding mix (ThermoScientific). Primers were all designed with Primer-BLAST (NCBI) and are listed in Table EV4D. qPCRs were produced using the SsoFast EvaGreen supermix (Bio-Rad). Expression was normalised with Actin primers and represented as fold change using the $2^{-\Delta\Delta CT}$ formula. For the identification of epithelial or mesenchymal subtypes in pancreatic murine tumour cells, R211 has been defined as the reference cell line.

Cytokine measurement in cell supernatants (Q-Plex™ assay)

The assay was performed according to the manufacturer's protocol. A Q-Plex™ assay (Quansys Biosciences, Q-Plex mouse cytokine Stripwells 16-plex assay) was carried out to measure the

concentration of 16 mouse cytokines in cell supernatants. The concentration was normalised with the number of living cells. R211, A338 and A260 were derived from primary tumours. A338L and A94L were isolated from lung metastases. All cell lines were passaged at least 15 times to avoid stromal contamination. KC;p110α$^{+/+}$ (R211, A338, A338L) and KC;p110α$^{+/lox}$ (A260, A94L) cells (3.10^{6}) were seeded in 6-well plates with 10% FBS. On the following day, the medium was replaced by 2% FBS medium with or without the vehicle (0.01% DMSO) or 1 µM BYL-719. After 2 days of treatment, the medium was replaced by a new medium with the same content. The cell supernatant was extracted after one day.

Detailed PIP extraction and measurement by mass spectrometry

Murine pancreatic tumour cells R211 were treated with or without the vehicle (0.01% DMSO), A66 1 µM or BKM120 1 µM for 15 minutes. The culture medium was quickly aspirated and cells were scraped in cold HCl 1M. The lysate was centrifuged for 5 minutes at 15,000 g at 4°C. The cell pellet was stored at -80°C until required for PIP extraction. Lipids were extracted and derivatised using TMS-diazo-methane as previously described (Clark *et al*, 2011). Mass spectral analysis was performed on the LC-QqQ triple quadrupole mass spectrometer (LC-vcQQQ 6460 Agilent) equipped with positive mode electrospray ionisation as described above (Clark *et al*, 2011). Analyses were performed in Selected Reaction Monitoring detection mode (SRM) using nitrogen as collision gas. Finally, peak detection, integration and quantitative analysis were performed using MassHunter QqQ Quantitative analysis software (Agilent Technologies VersionB.05.00) and Microsoft Excel software. Data were processed using QqQ Quantitative (vB.05.00) and Qualitative analysis software (vB.04.00).

Details of the IHC procedure (human and murine samples)

Tissues were fixed in 10% neutral-buffered formalin (Sigma HT501128) and embedded in paraffin. For pathological analysis, the tissues were serially sectioned (4 µm), and then stained with haematoxylin eosin. All tissues were analysed in blinded fashion. Histopathological scoring of pancreatic lesions was performed on sections 100 µm apart, with 3 sections per pancreas. IHC was performed and quantified as described in (Baer *et al*, 2014).

Immunostaining was conducted on formalin-fixed, paraffin-embedded tissues using standard methods with listed antibodies (Table EV4E). After rehydration, slides were permeabilised for 10 min in 0.1% Triton/1% PBS; they were then subjected to heat-antigen retrieval in citrate buffer or to enzymatic antigen retrieval in Proteinase K (Dako S3020), the latter was only applied for F4/80 immunostaining. Slides were incubated for 20 min with Protein Block (Dako X0909) to prevent non-specific binding. Sections were subsequently incubated overnight or for 1 h at room temperature* with primary antibodies (Table EV4E), washed, incubated for 15 min with 3% H_2O_2 and then rewashed. Most antibodies were detected using the HRP-Detection Reagent SignalStain®Boost (CST 8114) and developed through AEC (Dako K3464) incubation; anti-F4/80 antibody was revealed using the VECTASTAIN® Elite® ABC HRP Kit (VECTOR Laboratories PK-6100). All slides were counterstained with haematoxylin and mounted using Glycergel Mounting Medium (Dako C0563). Quantifications were randomly performed in at least 5 large-scale images in the area indicated.

We followed the manufacturers' protocol for additional staining with the Picro Sirius Red Stain Kit (ab150681) and Movat's Pentachrome Stain Kit (Modified Russell-Movat ab245884).

Appendix supplemental references

Baer R, Cintas C, Dufresne M, Cassant-Sourdy S, Schönhuber N, Planque L, Lulka H, Couderc B, Bousquet C, Garmy-Susini B, *et al* (2014) Pancreatic cell plasticity and cancer initiation induced by oncogenic Kras is completely dependent on wild-type PI 3-kinase p110α. *Genes Dev* 28: 2621–2635

Clark J, Anderson KE, Juvin V, Smith TS, Karpe F, Wakelam MJO, Stephens LR & Hawkins PT (2011) Quantification of PtdInsP3 molecular species in cells and tissues by mass spectrometry. *Nat Methods* 8: 267–272

Hingorani SR, Wang L, Multani AS, Combs C, Deramaudt TB, Hruban RH, Rustgi AK, Chang S & Tuveson DA (2005) Trp53R172H and KrasG12D cooperate to promote chromosomal instability and widely metastatic pancreatic ductal adenocarcinoma in mice. *Cancer cell* 7: 469–83

Therville N, Arcucci S, Vertut A, Ramos-Delgado F, Da Mota DF, Dufresne M, Basset C & Guillermet-Guibert J (2019) Experimental pancreatic cancer develops in soft pancreas: novel leads for an individualized diagnosis by ultrafast elasticity imaging. *Theranostics* 9: 6369–6379
